# Insights into the Mechanisms and Functional Effects of Insoluble Dietary Fiber Modification: A Review

**DOI:** 10.3390/foods15010038

**Published:** 2025-12-23

**Authors:** Jiayi Li, Wenjing Lang, Shuo Han, Xinyi Wu, Fuwei Hao, Yurong Zhou, Renpeng Du, Chen Song

**Affiliations:** Engineering Research Center of Agricultural Microbiology Technology, Ministry of Education & Heilongjiang Provincial Key Laboratory of Plant Genetic Engineering and Biological Fermentation Engineering for Cold Region & Key Laboratory of Microbiology, College of Heilongjiang Province & School of Life Sciences, Heilongjiang University, Harbin 150080, China

**Keywords:** insoluble dietary fiber, modification technology, functional properties, food applications

## Abstract

Dietary fiber is an essential component of the human diet, and insoluble dietary fiber (IDF) accounts for a significant proportion. However, its poor solubility and rigid structure limit its high-value applications. In recent years, modification technologies have become key strategies for enhancing the functional properties of IDF and expanding its applications. This review systematically summarizes the latest advances in the field of IDF modification, emphasizing how different modification strategies precisely regulate the multilevel structure of IDF to selectively improve its physicochemical properties and physiological functions. The principles and mechanisms of physical, chemical, biological, and combined modification methods are explained, and the unique advantages and limitations of each method in terms of structural changes, functional enhancement, and application scenarios are compared. Using high-pressure hydrostatic pressure-assisted cellulase treatment on potato dietary fiber can effectively disrupt fiber rigidity, increase soluble dietary fiber (SDF), and markedly enhance cholesterol and glucose adsorption capacities, outperforming single-treatment approaches. Microwave-assisted enzymatic treatment of millet bran IDF raises its intestinal fermentation rate from 36% to 59% and doubles butyrate production, significantly boosting prebiotic activity and offering a new pathway for targeted modulation of gut microbiota; combined modification strategies further demonstrate synergistic benefits. Modified IDF can serve not only as a low-calorie fat replacer in foods but also, through specific structural alterations, be incorporated into plant-based meat products to improve their fiber attributes and nutritional density. Moreover, this review explores the emerging potential of modified IDF in pharmaceutical carriers and gut microecology regulation. The aim is to provide theoretical guidance for selecting and optimizing IDF modification strategies, thereby promoting the high-value utilization of agricultural processing by-products and the development of high-quality dietary fiber products.

## 1. Introduction

In the modern field of food and nutrition science, insoluble dietary fiber (IDF) has attracted considerable attention due to its unique physicochemical properties and physiological functions. Although it is not easily digested and absorbed by the human body, it can increase fecal bulk and promote intestinal peristalsis, thereby providing clear benefits for preventing constipation and maintaining gut health [1]. However, the inherent rigid structure of IDF and its limited water-solubility and fermentability severely restrict its widespread application in refined food formulations and pharmaceutical carriers. To overcome these limitations, a variety of physical, chemical, and biological modification techniques have been developed to regulate the structure of IDF, aiming to enhance its functional properties and expand its application scope.

Modification technologies for insoluble dietary fiber (IDF) aim to overcome application bottlenecks by regulating its multi-scale structure. Physical modification technologies, such as high hydrostatic pressure and high-pressure homogenization, can disrupt the crystalline regions of fibers through intense mechanical forces, increase the specific surface area, and thereby improve their hydration properties. Among chemical modification methods, technologies like esterification alter the surface properties and reactivity of fibers by introducing hydrophobic groups [2]. In contrast, biological modification mainly utilizes cellulase and other enzyme preparations to specifically cleave the glycosidic bonds of fiber molecules, reduce their degree of polymerization, and may concomitantly produce oligosaccharides with prebiotic properties [3].

Despite the diversity of modification technologies, their efficacy varies significantly across different studies and fiber sources, which constitutes one of the core challenges in current research. Treatment of potato fiber with high hydrostatic pressure-assisted cellulase can synergistically promote the conversion of insoluble fiber to soluble fractions, increasing the soluble dietary fiber (SDF) content by up to 63.37% and significantly enhancing its adsorption capacities for cholesterol and glucose (by 92.70% and 50.70%, respectively) [3]. However, due to differences in their initial structures (e.g., crystallinity, porosity, and component ratios), IDF from different sources exhibit varying responses to the same modification technology, making it difficult to predict and standardize the modification effects. Furthermore, the current understanding of how modification precisely influences the fermentability of IDF in the gut and its interaction mechanisms with specific gut microbiota remains insufficiently in-depth.

This review aims to systematically summarize the latest advances in the major modification technologies for insoluble dietary fiber (IDF), with a particular focus on the structure-activity relationships between the structural changes induced by different technologies and the corresponding functional enhancements. It will conduct an in-depth analysis of the inconsistent results reported in current studies and explore the potential underlying reasons, such as the heterogeneity of fiber sources and subtle differences in modification conditions. Meanwhile, this review will clarify common misunderstandings regarding the physiological function mechanisms of IDF (e.g., not all fibers can effectively alleviate constipation, as their efficacy depends on the fiber type and physical form) and identify the existing knowledge gaps in current research regarding mechanism elucidation and large-scale technological application. This work intends to provide a theoretical reference for the high-value utilization of IDF and the direction of future research.

## 2. Definition and Classification of Dietary Fiber

Dietary Fiber (DF) refers to a class of polysaccharide substances that cannot be digested and absorbed in the human small intestine. Based on its solubility, it can be classified into Soluble Dietary Fiber (SDF) and Insoluble Dietary Fiber (IDF). While the two exhibit significant differences in structure and function, they collectively play a crucial role in regulating physiological health, thereby having been recognized as “the seventh nutrient” [4].

Soluble Dietary Fiber (SDF), such as pectin, inulin, and β-glucan, is mainly derived from plant cell walls and exhibits excellent water solubility and gelling properties [5]. It is fermented by gut microbiota in the colon to produce short-chain fatty acids (SCFAs), thereby exerting prebiotic properties and participating in the regulation of blood glucose, blood lipids, and inflammatory responses.

Insoluble Dietary Fiber (IDF), on the other hand, is primarily composed of cellulose, hemicellulose, and lignin. It is insoluble in water and possesses a stable physicochemical structure [6]. Cellulose is a linear glucose polymer linked by β-1,4-glycosidic bonds; hemicellulose is a heteropolysaccharide containing multiple monosaccharide units with a branched structure; and lignin is a three-dimensional network polymer composed of phenylpropane derivatives [7].

By virtue of its surface active groups, IDF adsorbs lipid and carbohydrate molecules in the intestinal tract, enhances satiety, promotes intestinal peristalsis, and plays a key role in metabolic regulation [8]. Understanding the structural and functional differences between SDF and IDF lays the foundation for further research on their modification technologies and applications.

### 2.1. Health-Promoting Effects of Soluble Dietary Fiber (SDF)

Soluble Dietary Fiber (SDF), such as β-glucan, pectin, and inulin, can dissolve in water to form a viscous gel, thereby significantly delaying gastric emptying and the absorption of glucose in the small intestine, which helps stabilize postprandial blood glucose responses [9]. Meanwhile, SDF can bind to bile acids, promoting their excretion in feces and compelling the liver to utilize more cholesterol for de novo bile acid synthesis. This mechanism effectively reduces levels of total serum cholesterol (TSC) and low-density lipoprotein cholesterol (LDL-C). More importantly, SDF serves as the primary nutrient source for beneficial gutmicrobiota (e.g., Bifidobacteria and Lactobacilli) in the colon. A large amount of short-chain fatty acids (SCFAs) produced by microbial fermentation of SDF—particularly butyrate—not only acts as an energy source for colonic epithelial cells and maintains intestinal barrier integrity but also enters the circulatory system to exert systemic anti-inflammatory and immunomodulatory effects [10].

### 2.2. Health-Promoting Effects of Insoluble Dietary Fiber (IDF)

Insoluble Dietary Fiber (IDF), such as cellulose, partial hemicellulose, and lignin, is defined by core functions derived from its strong water-holding capacity and resistance to fermentation. Upon absorbing water in the intestinal tract, it swells, significantly increasing fecal volume and softening feces while directly stimulating the intestinal wall and accelerating intestinal peristalsis—thereby effectively preventing and alleviating constipation. Furthermore, the expanded fecal volume can dilute potential carcinogens in the intestinal lumen and shorten their contact time with the intestinal mucosa. Additionally, the physical network formed by IDF exerts a physical entrapment effect on substances, including glucose, bile acids, and lipids, in the intestine. Although its hypoglycemic and hypocholesterolemic effects are inferior to those of SDF, IDF still holds important auxiliary significance in metabolic regulation.

### 2.3. Synergistic Effects & Modification Goals

The health-promoting effects of SDF and IDF are not isolated; they are complementary. A healthy dietary pattern requires the coexistence of both. However, natural food raw materials often contain IDF with dense structures and rough textures, which limit their application in food products and the exertion of their physiological functions. This is precisely the core objective of IDF modification technology: utilizing physical, chemical, or biological means to specifically disrupt the rigid structure of IDF. On one hand, this improves its processing and sensory properties; on the other hand, it activates and enhances its inherent health-promoting functions. Furthermore, by converting part of the IDF into SDF or creating porous structures to enhance adsorption capacity, we can achieve a “1 + 1 > 2” synergistic effect on health.

## 3. Overview of Dietary Fiber Modification Technology

### 3.1. Physical Modification Technology

#### 3.1.1. Heat Treatment

Heat treatment is a widely applied physical modification technique that has garnered significant attention in the research and application of insoluble dietary fibers (IDFs). By regulating parameters such as temperature, time, and heating methods, it can specifically affect the structures of cellulose and hemicellulose within IDFs: thermal action disrupts the intra- and inter-molecular hydrogen bond networks of cellulose, reducing its crystallinity by 10–20%, while causing depolymerization and a decrease in molecular weight. Hemicellulose, due to its amorphous structure and heteropolysaccharide characteristics, is more sensitive to heat, undergoing glycosidic bond cleavage and chain degradation; its average molecular weight can drop by 30–50%, resulting in shorter chain lengths. After structural depolymerization, both components expose more active sites on their surfaces, enhancing interactions with lipids, proteins, and other nutrients. Consequently, this technology holds significant potential for improving the functional properties of dietary fibers in the field of functional foods.

For example, Dong et al. [11] investigated the effects of four heat treatment methods—atmospheric pressure cooking (100 °C, 20 min), high-pressure cooking (0.1 MPa, 121 °C, 20 min), microwave treatment (700 W, 3 min), and deep-frying (180 °C, 5 min)—on the structural and functional properties of soluble dietary fiber (SDF) in whole-grain oats. The results showed that heat treatment significantly increased the contents of total dietary fiber (TDF), insoluble dietary fiber (IDF), and soluble dietary fiber (SDF) in oats, while also enhancing the molecular weight, particle size, and aggregation degree of SDF.

Ai et al. [12] investigated the effects of three heat treatment methods—atmospheric boiling (AB), high-pressure boiling (HPB), and baking (B)—on the structural, physicochemical, and functional properties of dietary fiber (DF) in whole-grain highland barley (HB). The results indicated that heat treatment caused the cleavage of 3,4-linked rhamnopyranose and 2-linked mannopyranose in soluble dietary fiber (SDF), leading to a decrease in molecular weight and reductions in the proportions of rhamnose and mannose. Compared with untreated highland barley, the molar ratios of glucose in SDF from AB-treated HB (AB-HB), HPB-treated HB (HPB-HB), and baked HB (B-HB) increased by 53.18%, 51.52%, and 47.75%, respectively. Heat treatment reduced the apparent viscosity, storage modulus (G′), and loss modulus (G″) of insoluble dietary fiber (IDF), decreased the particle size of DF, and increased its specific surface area—thereby enhancing the adsorption capacities of DF (including glucose adsorption capacity, cholesterol adsorption capacity, and nitrite adsorption capacity). Additionally, heat treatment improved the antioxidant activity of DF and its inhibitory capacity against pancreatic lipase; specifically, HPB and baking treatments enhanced the inhibitory effects of SDF on α-amylase and α-glucosidase, as well as the inhibitory effect of IDF on α-glucosidase.

In recent years, studies on IDF modification using individual heat treatments have yielded promising results; however, their overall effectiveness remains limited compared to that of combined treatment approaches. This method is mainly used for basic modification in the food industry, while the composite treatment has become the focus of research and industrial application due to its more significant effect and comprehensive function. In the future, with growing demands for environmental sustainability and processing efficiency, individual heat treatments may be integrated with other technologies to further optimize the functional properties of IDF.

#### 3.1.2. Microwave Radiation

As a novel physical modification technology, microwave radiation has gradually demonstrated unique advantages in the modification of insoluble dietary fiber (IDF). Compared with traditional heating methods, microwave radiation achieves rapid and uniform heating of substances through electromagnetic waves, thereby altering the structural and functional properties of dietary fiber. By adjusting microwave power and treatment time, the modification effect of dietary fiber can be precisely regulated to enhance its functional properties [13,14]. Microwave radiation exerts a significant impact on IDF modification: microwave heating can raise the temperature of dietary fiber to the target temperature in a short time, avoiding the local overheating issue commonly encountered in traditional thermal processing, which is more conducive to retaining its nutritional components.

For example, Cantu-Jungles et al. [15] modified insoluble dietary fiber (IDF) extracted from corn stover using microwave radiation and investigated its effects on the physicochemical properties of the fiber and the fermentation capacity of gut microbiota. The results showed that microwave radiation significantly enhanced the hydration capacity and solubility of dietary fiber. When the microwave power was 700 W and the treatment time was 5 min, the water-holding capacity (WHC) of IDF increased from 3.23 g/g to 5.41 g/g, representing an increase of approximately 67.5%; the swelling capacity increased from 4.25 mL/g to 7.30 mL/g, with a growth rate of about 71.8%. More importantly, the modified IDF exhibited stronger prebiotic potential, as it could more effectively promote the growth of beneficial gut bacteria (e.g., Bifidobacteria and Bacteroides) and stimulate the production of higher levels of short-chain fatty acids (SCFAs).

Lei et al. [16] explored the effects of microwave radiation treatment on insoluble dietary fiber (IDF) from black soybean dregs. The results indicated that microwave radiation disrupts the dense structure of dietary fiber through thermal and mechanical effects, increases its porous space and specific surface area, and significantly enhances its water-holding capacity (WHC), swelling power, and antioxidant activity. Compared with traditional heat treatment (e.g., cooking), microwave radiation is more effective in destroying the crystalline structure of dietary fiber: its crystallinity decreased from 34.6% to 25.3%, a reduction of approximately 26.9%. Additionally, due to the short treatment duration, microwave radiation can better retain the chemical components of dietary fiber and avoid the loss of nutritional components caused by excessive heating. Although microwave radiation can effectively improve the functional properties of IDF in laboratory settings, its industrial application is still limited to basic modification and needs to be combined with other technologies to achieve more efficient and targeted structural regulation.

#### 3.1.3. Ultrasonic Treatment

As an efficient physical modification technology, ultrasonic treatment can precisely regulate the microstructure of insoluble dietary fiber (IDF) through the synergistic effect of cavitation and mechanical shear force, thereby improving its functional properties. Under appropriate ultrasonic conditions (e.g., power: 500–600 W, time: 30–45 min, temperature: 40–50 °C), the intense formation and collapse of cavitation bubbles generate local high temperature and pressure, which disrupt the hydrogen bond network and crystalline regions of cellulose. Meanwhile, it simultaneously promotes the formation of micropores and cracks on the fiber surface, significantly increasing the specific surface area. Mechanical shear force further facilitates fiber fragmentation and dispersion, enhancing the contact efficiency between fibers and the aqueous phase [16,17].

For example, Huang et al. modified insoluble dietary fiber (IDF) from garlic straw using wet ultrasonic treatment. Through the uniform design (UD) method, the optimal ultrasonic conditions for preparing IDF with excellent in vitro hypolipidemic capacity were identified as an initial temperature of 45 °C, ultrasonic power of 535 W, and treatment time of 41 min. The results showed that the ultrasonically treated IDF was superior to the untreated IDF in both functional and physicochemical properties [18]. Kalla-Bertholdt A M et al. investigated the effects of ultrasonic treatment on the functional properties of citrus fiber, apple fiber, oat fiber, and pea fiber. The results indicated that ultrasonic treatment could significantly improve the water-holding capacity (WHC) of citrus fiber and apple fiber [19].

Although ultrasonic treatment exhibits excellent modification efficiency and controllability in laboratory-scale applications, its industrial scaling-up still faces challenges such as processing uniformity, energy consumption, and economic feasibility. Future research could focus on combining ultrasound with technologies like enzymatic methods and microwave radiation to achieve multi-level precise regulation of the structure and functions of IDF, thereby promoting its innovative applications in functional foods.

#### 3.1.4. High-Pressure Homogenization Modification

High-pressure homogenization (HPH) is a non-thermal processing technology. This technology enables fluid materials to pass through narrow gaps at high speed under high pressure (typically 100–200 MPa), generating the synergistic effect of intense shear, turbulence, impact, and cavitation, which can precisely deconstruct the macro- and micro-morphologies of insoluble dietary fiber (IDF) [20]. The core mechanism of this technology lies in its strong mechanical forces, which can effectively break fiber bundles, disrupt cell wall structures, and induce the physical depolymerization of cellulose and hemicellulose. This directly results in two key structural changes: a significant reduction in the particle size of IDF and the formation of a porous, loose microstructure. These structural modifications serve as the physical basis for the enhanced functional properties of IDF via HPH treatment. At the functional level, HPH treatment imparts multiple improvements to IDF, with its main functional outcomes summarized as follows: Enhanced hydration properties: Due to the increased specific surface area and exposure of hydrophilic groups, the water-holding capacity (WHC), swelling capacity (SWC), and oil-holding capacity (OHC) of HPH-treated IDF are generally significantly enhanced. Improved emulsifying properties: The reduced particle size and increased surface activity enable modified IDF to effectively adsorb at the oil-water interface and stabilize Pickering emulsions, thereby improving the emulsifying performance and stability of food systems [21]. Increased soluble fractions: Mechanical forces can promote the cleavage or dissolution of partially insoluble polysaccharides, thereby increasing the yield of soluble dietary fiber (SDF) and optimizing the overall composition of dietary fiber.

For example, Yang et al. [22] investigated the effects of high-pressure homogenization (HPH) treatment on the emulsifying properties and stability of citrus insoluble dietary fiber (IDF). The results showed that HPH treatment improved the emulsifying properties of citrus IDF: both 100 MPa and 200 MPa pressure treatments reduced the particle size of citrus IDF, promoted its interfacial adsorption and network formation in the continuous phase, and thereby enhanced the emulsifying performance and stability of Pickering emulsions. Wang et al. [23] studied the effects of HPH modification on the functional properties of litchi pulp IDF. The results indicated that HPH altered the microstructure of litchi pulp IDF, resulting in a more porous structure. Consequently, its water-holding capacity (WHC), water absorption capacity, swelling capacity, and oil-holding capacity (OHC) were significantly enhanced, while the total phenolic content and antioxidant activity also moderately increased.

Although high-pressure homogenization (HPH) has demonstrated excellent potential for insoluble dietary fiber (IDF) modification in laboratory studies, its industrial scaling-up still faces challenges related to energy consumption and equipment costs. Future research will focus more on optimizing homogenization parameters (e.g., pressure, cycle times) and exploring their synergistic effects with technologies such as enzymatic methods or ultrasonic treatment, with the aim of achieving multi-level precise regulation of the structure and functions of IDF and promoting its high-value applications in functional foods.

#### 3.1.5. High Hydrostatic Pressure

High hydrostatic pressure (HHP) is an efficient physical modification technology, particularly suitable for high-fiber materials such as fruit peels and cereal by-products. By applying isotropic hydrostatic pressure to insoluble dietary fiber (IDF) systems within a specific pressure range (typically 100–600 MPa), HHP achieves precise physical modification of fiber structures. Studies have shown that due to its operating conditions at room temperature or mild heating, this technology can effectively retain thermosensitive components while avoiding the use of chemical reagents, making it recognized as an efficient and environmentally friendly processing method [24]. At the structural level, HHP treatment acts on IDF through two main mechanisms: Macrostructural deconstruction: The instantaneous application of high pressure to the fiber-water system generates a pressure difference that disrupts the integrity of plant cell walls, loosens the three-dimensional network structure of cellulose-hemicellulose-lignin, and results in fiber fragmentation and reduced particle size. Molecular-level structural changes: The intense hydrostatic pressure can break non-covalent bonds (e.g., hydrogen bonds and hydrophobic interactions) that maintain fiber structure, and even cleavage of glycosidic bonds has been observed in some studies. This directly leads to a decrease in the crystallinity of cellulose microfibrils and exposes previously embedded active sites. These structural modifications are directly associated with enhanced functional properties of IDF, with key improvements summarized as follows: Increased SDF yield: Deconstruction of the fiber network promotes the release of more soluble fractions, and studies have reported a 15–30% increase in SDF content. Enhanced hydration properties: Structural loosening and increased specific surface area significantly improve water-holding capacity (WHC) and swelling capacity (SWC). Improved bioactive potential: Exposure of active sites enhances antioxidant activity and adsorption capacity for glucose and cholate.

In summary, by precisely controlling pressure parameters, HHP technology can directionally regulate the degree of IDF structural deconstruction, thereby achieving precise optimization of its physicochemical properties and physiological activities [25]. As a non-thermal modification technology, it exhibits great potential in the development of high-value-added dietary fiber ingredients. Notably, it enhances functional properties while retaining the activity of natural components, providing a novel technical pathway for the innovation of functional foods.

**Figure 1:**
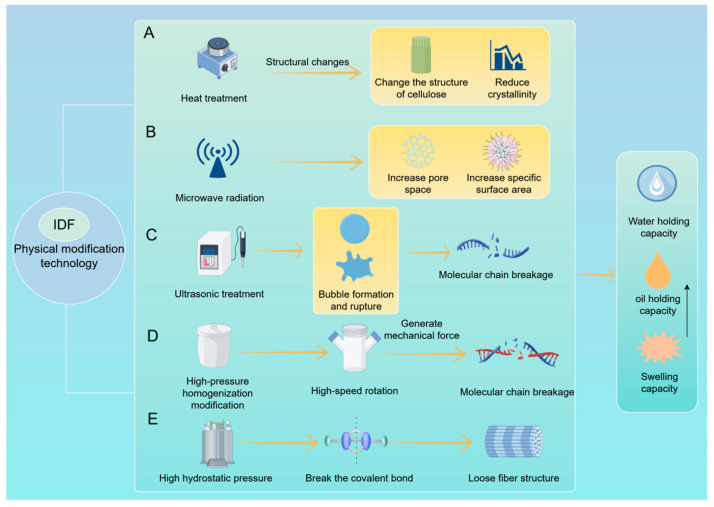
The mechanism of physical modification. (**A**). Heat treatment; (**B**). Microwave radiation; (**C**). Ultrasonic treatment; (**D**). High-pressure homogenization modification; (**E**). High hydrostatic pressure. (↑ An upward arrow represents an enhancement symbol).

In short, physical modification methods such as heat treatment, microwave treatment, ultrasonic treatment, and high-pressure treatment can change the paracellular structure of dietary fiber, destroy the cellulose alert zone, increase the surface area, and improve the water and oil holding capacity (Figure 1).

#### 3.1.6. Micronization Technology

Micronization technology is a physical modification method that crushes material particles to the micron or even nanometer scale via mechanical forces. Common micronization techniques include high-pressure micronization, jet milling, and ultra-fine grinding. This technology exhibits a universal regularity in disrupting the structure, increasing porosity, and improving water solubility of insoluble dietary fiber (IDF) from various sources, with reduced particle size and exposed hydrophilic groups identified as the key factors for enhanced functionality [26]. Micronization can effectively destroy the dense crystalline structure of IDF, exposing previously encapsulated hydrophilic groups such as hydroxyl groups, thereby significantly enhancing its hydration capacity (e.g., water-holding capacity (WHC), swelling capacity (SWC), and water solubility). The efficacy of micronization modification is closely related to particle size: as particle size decreases, the specific surface area of IDF undergoes a sharp increase. This not only strengthens its interaction with water molecules but also improves its adsorption capacity for harmful substances such as cholesterol, bile acids, and heavy metals, thereby enhancing its physiological health functions.

Additionally, ultra-fine dietary fiber powder possesses superior dispersibility and mouthfeel, enabling it to be more uniformly incorporated into various food systems (e.g., beverages, baked goods, and meat products). It improves the texture of products without introducing a coarse granular sensation.

However, micronization technology also has certain limitations. Excessive micronization may cause excessive cleavage of dietary fiber molecular chains and a decrease in molecular weight, which, conversely, impairs its gel-forming capacity and partial physiological activities. In addition, this process is associated with high energy consumption and strict equipment requirements. Therefore, optimizing the grinding process and controlling an appropriate particle size range are the keys to the successful application of micronization technology in insoluble dietary fiber (IDF) modification. In the future, combining micronization with other modification technologies (e.g., enzymatic hydrolysis, chemical modification) is expected to achieve synergistic enhancement of functional properties, further expanding the application of IDF in the high-end functional food sector.

### 3.2. Chemical Modification Technology

#### 3.2.1. Esterification Modification

Esterification modification is a chemical technology that modifies the molecular structure of insoluble dietary fiber (IDF) by introducing ester functional groups, enabling significant regulation of its physicochemical properties. This method typically utilizes esterifying agents such as organic acids or acid anhydrides, which undergo a reaction with the hydroxyl groups in the IDF structure to form ester bonds, thereby altering the polarity and intermolecular interactions of the fiber [27].

The core of esterification modification lies in regulating the hydrophilic-hydrophobic balance of IDF by altering its degree of substitution (DS). With the increase in DS, the number of ester groups introduced onto the IDF molecular chains increases. As polar functional groups, these ester groups can enhance the hydrogen bonding between fibers and water molecules, thereby improving hydrophilicity and swelling capacity. However, the improvement in hydrophilicity is not linearly dependent on DS: at low DS, the newly introduced ester groups mainly disrupt the crystalline regions of cellulose, making the molecular chains more susceptible to hydration and thus significantly enhancing water-holding capacity (WHC) and swelling capacity; at high DS, excessive hydrophobic ester groups may induce a steric hindrance effect, which conversely weakens its hydrophilic properties to some extent but may simultaneously strengthen its hydrophobic interactions with non-polar substances (e.g., cholesterol) [28].

The effects of esterification modification on the various functions of IDF are not unidirectional, which is directly related to its mechanism of action. For instance, the introduced hydrophobic acetyl groups can bind to cholesterol molecules through hydrophobic interactions, so the cholesterol adsorption capacity of modified IDF generally increases with increasing DS. In contrast, antioxidant activity decreases: the esterification reaction consumes some antioxidant active groups (e.g., natural phenolic hydroxyl groups) in the IDF structure, or reduces the free radical scavenging capacity due to steric hindrance, leading to a decrease in overall antioxidant activity [29]. Products with high DS exhibit better performance in inhibiting α-amylase activity due to their superior solubility and steric hindrance effect on digestive enzymes.

For example, Ke et al. [29] modified wheat bran insoluble dietary fiber (IDF) via acetylation using acetic anhydride, systematically investigating the effects of degree of substitution (DS) on the fiber’s structure and functions. The results showed that acetylation significantly enhanced the cholesterol adsorption capacity and in vitro hypoglycemic activity (e.g., α-amylase inhibitory activity) of IDF: the cholesterol binding capacity increased from 6.21 mg/g in unmodified samples to 11.35 mg/g in high-DS samples; the α-amylase inhibition rate also significantly increased from 15.2% to 42.7%. Meanwhile, acetylation reduced the crystallinity and antioxidant activity of IDF. The study also found that products with low DS exhibited higher water-holding capacity (WHC), while those with high DS showed better hypoglycemic performance. Compared with unmodified dietary fiber, both the solubility and swelling capacity of esterified modified fiber were improved, making its application effects more prominent in functional foods.

#### 3.2.2. Oxidation Modification

Oxidation modification is a chemical modification technology that introduces oxygen-containing functional groups (e.g., carboxyl groups, carbonyl groups) into the molecular structure of insoluble dietary fiber (IDF) through the action of oxidizing agents (e.g., hydrogen peroxide, hypochlorites). This process breaks the hydrogen bonds and glycosidic bonds of cellulose, disrupts its crystalline regions, and reduces the degree of polymerization (DP), thereby significantly altering the physicochemical properties of IDF. Among various oxidation methods, alkaline hydrogen peroxide (AHP) treatment is regarded as a relatively environmentally friendly modification approach, as its only reaction by-products are water and oxygen [30].

The core mechanism of oxidation modification lies in the introduction of polar oxygen-containing functional groups onto IDF molecular chains via oxidation reactions. The incorporation of carboxyl groups and other polar groups significantly enhances the hydrophilicity and ion exchange capacity (IEC) of the fiber. Simultaneously, the cleavage of molecular chains and the decrease in crystallinity render the fiber structure looser and more porous, with an increased specific surface area. These structural changes collectively contribute to the general improvement of IDF’s hydration properties (e.g., water-holding capacity (WHC), swelling capacity (SWC)) and solubility [28].

Studies have shown that after AHP treatment, the water-holding capacity (WHC) and swelling capacity (SWC) of IDF can generally be increased by 30% to 45%. Notably, the effects of oxidation modification on the various functions of IDF are not uniform. While enhancing hydration properties and adsorption capacity (e.g., adsorption of cholesterol, glucose, and cholate), this process may also degrade some coexisting natural phenolic compounds (e.g., isoquercitrin, ferulic acid), thereby leading to a decrease in overall antioxidant activity [31]. This functional discrepancy mainly stems from two aspects: the newly introduced carboxyl groups enhance the binding capacity with polar substances; the oxidative environment causes structural damage to oxidation-sensitive phenolic compounds. Although hydrogen peroxide decomposes easily after use, in food industrial applications, strict control of oxidant residues is required, and attention should be paid to potential adverse by-products that may be generated due to excessive oxidation [32]. Furthermore, random cleavage of glycosidic bonds during modification may produce indigestible small-molecule fragments, whose long-term physiological effects need further evaluation. Regulatory authorities worldwide (e.g., National Health Commission of the People’s Republic of China (NHC), FDA, EFSA) have established strict limits and regulatory requirements for the use of oxidants in food processing, and compliance with relevant regulations must be ensured prior to practical application.

For example, M. et al. [33] optimized the bleaching process of wheat bran using hydrogen peroxide (H_2_O_2_) via response surface methodology (RSM) and evaluated its effects on wheat bran properties. The study found that under optimal conditions (H_2_O_2_ concentration: 1.5%, temperature: 50 °C, time: 60 min), the whiteness value of wheat bran significantly increased by 46.7%, while its water-holding capacity (WHC) improved from 2.85 g/g to 3.51 g/g, representing an increase of 23.2%. These results indicate that the functional properties of wheat bran were effectively enhanced. Similarly, studies by Grzelczyk et al. [34] and Stryjecka et al. [35] have also shown that hydrogen peroxide treatment can effectively alter the structural and functional properties of dietary fiber. The results demonstrated that the water absorption capacity and solubility of modified fiber were significantly improved: the swelling capacity (SWC) of oxidized dietary fiber increased by 35%, and the water absorption capacity was enhanced by 45%. Additionally, oxidation treatment induced molecular chain relaxation of dietary fiber, which improved its hydration capacity and swelling properties, further enhancing the application potential of dietary fiber in food processing.

In summary, oxidation modification is an effective approach to improving the functional properties of insoluble dietary fiber (IDF), but it requires striking a balance between benefits and potential risks (e.g., loss of bioactive components and safety concerns). By precisely controlling parameters such as oxidant concentration, reaction pH, and temperature, the desired functionalities can be maximized while minimizing adverse effects.

**Figure 2:**
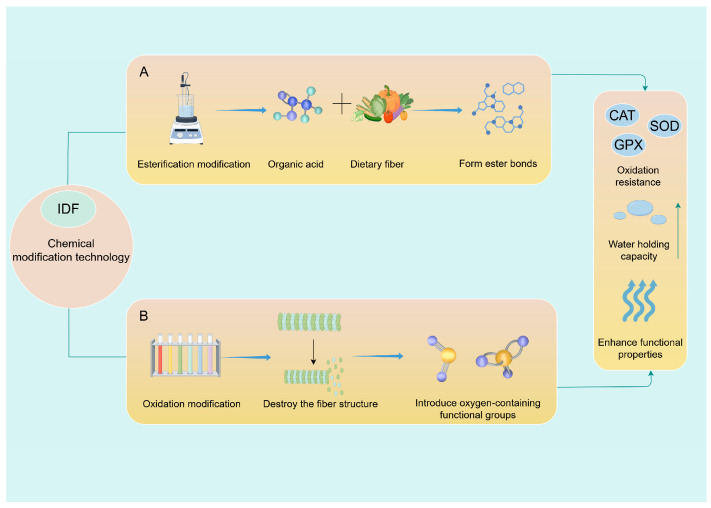
The mechanism of chemical modification. (**A**). Esterification modification; (**B**). Oxidation modification. (↑ An upward arrow represents an enhancement symbol).

In summary, chemical modification involves altering the functional groups of dietary fibers through specific chemical reactions. Techniques such as esterification, oxidation, and alkaline treatment introduce functional groups (e.g., carboxyl and ester groups) into the fiber structure, thereby reducing molecular weight, modifying hydrophilic or hydrophobic properties, and enhancing the binding capacity with functional components (Figure 2).

### 3.3. Biological Modification Technology

#### 3.3.1. Enzymatic Modification

Enzymatic modification is a green technology that utilizes specific enzyme preparations to perform biocatalysis on insoluble dietary fiber (IDF). It alters the physicochemical properties of IDF by targeted degradation of specific components in plant cell wall structures. The mechanisms of action of different enzymes exhibit high specificity: cellulase specifically hydrolyzes the β-1,4 glycosidic bonds in the cellulose backbone, directly reducing the degree of polymerization (DP) of the fiber; hemicellulases such as xylanase target the side chains of hemicellulose, disrupting the cross-linked structure of the fiber network; pectinase decomposes the pectin components in the intercellular matrix, inducing the loosening of the fiber structure [36]. The synergistic effect of such enzymatic hydrolysis collectively contributes to the transformation of IDF’s microstructural and macrostructural properties.

Numerous studies have confirmed that, despite variations in enzyme preparations and substrate sources, enzymatic modification generally induces a series of common structural changes, including increased surface porosity, decreased crystallinity, and expanded specific surface area [37]. These structural modifications form the physical basis for the functional enhancement of IDF. In terms of functional improvements, it has been found that the yield of soluble dietary fiber (SDF) and solubility are enhanced after enzymatic modification. Enzymatic hydrolysis cleaves the macromolecular network of IDF, promoting the conversion of partially insoluble components into SDF.

Jiang et al. [38] employed cellulase or xylanase for the single enzymatic modification of ginseng insoluble dietary fiber (IDF). Characterization by Fourier transform infrared spectroscopy (FTIR) and scanning electron microscopy (SEM) confirmed that enzyme treatment induced the formation of a porous and wrinkled surface structure on IDF, accompanied by a decrease in crystallinity. After modification, the water-holding capacity (WHC), swelling capacity, and cholesterol adsorption capacity of IDF increased by 32.6%, 29.8%, and 32.7%, respectively. Additionally, its nitrite adsorption capacity and α-amylase inhibitory activity were moderately enhanced. Furthermore, enzyme treatment improved the thermal stability of IDF, making it suitable for food processing below 300 °C. Liu et al. [39] treated rice bran IDF with a single cellulase. The results showed that enzymatic hydrolysis significantly reduced the contents of cellulose and hemicellulose while increasing the proportion of lignin. SEM observation revealed that enzyme treatment enhanced the surface porosity of IDF and exposed more binding sites. After modification, the WHC, oil-holding capacity (OHC), and glucose adsorption capacity of IDF increased by 21.3%, 18.6%, and 24.1%, respectively, whereas the emulsifying activity slightly decreased. This phenomenon may be attributed to the selective removal of some emulsification-active components by enzymatic hydrolysis.

These differential effects reveal the complexity of enzymatic modification: the direction and extent of changes in functional properties depend not only on enzyme specificity but also on the combined regulation of substrate composition, reaction conditions, and modification degree. Through the precise selection of enzyme types and control of hydrolysis extent, the functional properties of insoluble dietary fiber (IDF) can be directionally regulated to meet specific application requirements.

#### 3.3.2. Microbial Fermentation Modification

Microbial fermentation modification is a method that utilizes enzyme systems secreted by specific microorganisms (e.g., yeast, lactic acid bacteria (LAB), and fungi) to biodegrade insoluble dietary fiber (IDF) during fermentation, thereby altering its structural and functional properties. The core mechanism of this technology lies in the ability of different microbial strains to produce specific enzyme systems: fungi (e.g., *Aspergillus niger*, *Trichoderma reesei*) typically secrete abundant cellulase, hemicellulase, and pectinase, which can efficiently degrade the cellulose crystalline structure and hemicellulose network in plant cell walls; lactic acid bacteria (e.g., *Lactobacillus plantarum*, *Lactobacillus acidophilus*) mainly produce cellulase and hemicellulase with low to moderate activity, accompanied by the production of auxiliary enzyme systems such as esterases, which exert mild modification on the fiber surface [40]. These enzyme systems act synergistically to cleave the polysaccharide chains of IDF, reduce its molecular weight, and disrupt crystalline regions, thereby improving its solubility and swelling capacity.

Although microbial fermentation modification possesses biological advantages such as mild reaction conditions and endogenous enzyme system production, its industrial application still faces significant limitations. The fermentation process typically takes 24–72 h, which is much longer than that of physical or chemical modification; strain cultivation and fermentation control significantly increase production costs; meanwhile, strain variability and the high sensitivity of enzyme activity to fermentation conditions (e.g., pH, temperature, nutrients) result in considerable difficulty in controlling batch-to-batch stability [41].

Studies have shown that microbial fermentation modification not only alters the physical structure of insoluble dietary fiber (IDF) (e.g., forming a porous and wrinkled structure, reducing crystallinity) but also significantly enhances its functional properties. Compared with other physical and chemical modifications, microbial fermentation modification has distinct advantages: mild reaction conditions that do not require extreme parameters such as high temperature and high pressure, making it more effective in retaining the natural nutrients of dietary fiber. Table 1 lists the functional improvements and typical structural changes of SDF/IDF through microbial fermentation modification. Liao et al. [40] conducted 24 h fermentation modification of wheat bran IDF at 37 °C using single strains (*Lactobacillus plantarum*, *Lactobacillus acidophilus*, *Bacillus subtilis*) and mixed lactic acid bacteria (LAB). The results showed that after single fermentation with L. plantarum or mixed LAB fermentation, the water-holding capacity (WHC), oil-holding capacity (OHC), and swelling capacity of IDF increased by 21.3%, 18.6%, and 24.1%, respectively. Characterization by Fourier transform infrared spectroscopy (FTIR) and scanning electron microscopy (SEM) confirmed that fermentation induced the formation of a porous and wrinkled surface structure on IDF and reduced its crystallinity. Additionally, fermentation enhanced the nitrite adsorption capacity and total phenolic content of IDF, while its cholesterol adsorption capacity slightly decreased. Yang et al. [42] compared the modification effects of single-strain fermentation of black soybean okara IDF at 37 °C for 24 h using LAB and *Kluyveromyces marxianus*. The results indicated that single LAB fermentation increased the WHC and swelling capacity of IDF by 1.74-fold and 1.53-fold, respectively, with the formation of a honeycomb-like network structure on the surface. Fermentation significantly reduced the particle size and cellulose content of IDF, and the proportion of soluble dietary fiber (SDF) increased from 1.40% to 6.17%. Furthermore, the in vitro prebiotic activity of the single-fermentation groups was significantly enhanced, which could promote the proliferation of Bifidobacterium and LAB in the gut microbiota.

Notably, fermented modified dietary fiber not only exhibits improved solubility but also can be better utilized by gut microorganisms, promoting the growth of probiotics and enhancing intestinal health functions. Specifically, in vitro fermentation experiments have demonstrated that wheat bran IDF modified by *L. plantarum* fermentation can significantly promote the proliferation of beneficial gut bacteria (e.g., *Bacteroides* and *Bifidobacterium*) and produce more short-chain fatty acids (SCFAs), especially propionic acid and butyric acid, indicating a significant enhancement of its prebiotic activity.

In general, microbial fermentation modification can directionally improve the structural and functional properties of insoluble dietary fiber (IDF) via the synergistic effect of microbial enzyme systems. Although it has limitations such as long processing time, high production cost, and complex process control, it still possesses unique advantages and application potential in the development of functional food ingredients (e.g., probiotic foods, intestinal health-promoting products).

**Table 1:** Functional Improvement Effects and Typical Structural Changes in SDF/IDF via Microbial Fermentation Modification.

Microorganism Type	Main Enzyme System	Functional Improvement Effect	Typical Structural Change	Reference
*Lactic acid bacteria*	Cellulase, hemicellulase	The proportion of soluble dietary fiber (SDF) increased to 6.17%, and the water-holding capacity/swelling capacity improved by 1.5–1.7 times.	Porous wrinkled structure and decreased crystallinity.	[43]
*Lactobacillus plantarum*	Cellulase, hemicellulase	Water-holding capacity (WHC)/oil-holding capacity (OHC)/swelling capacity (SC) improved by 18–24%, and the phenolic content increased.	Surface porosification and decreased crystallinity.	[40]
*Kluyveromyces marxianus*	Cellulase, β-glucosidase	Enhanced prebiotic activity promotes the proliferation of Bifidobacterium/Lactobacillus.	Honeycomb-like network structure and decreased particle size.	[42]

**Figure 3:**
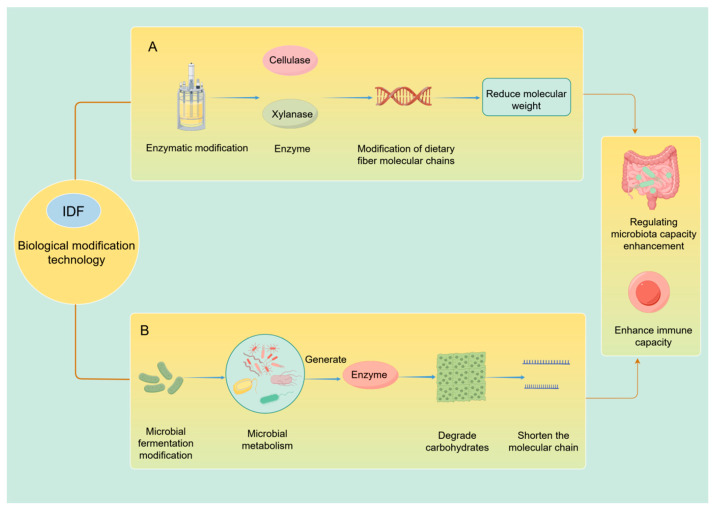
The mechanism of biological modification. (**A**). Enzymatic modification; (**B**). Microbial fermentation modification.

Overall, biomodification methods primarily include enzymatic treatment and microbial fermentation. These approaches enhance the prebiotic activity of insoluble dietary fibers by degrading cellulose and hemicellulose, improving solubility, and modifying molecular structures, particularly through the cleavage of long-chain polysaccharides (Figure 3).

### 3.4. Composite Modification Technology

Combined modification technology integrates multiple distinct modification methods for dietary fiber to achieve more desirable functional effects. The core of this strategy lies in using primary treatment to disrupt the macro- or micro-structure of insoluble dietary fiber (IDF), creating more favorable conditions for subsequent modification. This approach overcomes the limitations of single methods and achieves a synergistic effect of “1 + 1 > 2”. Studies have shown that combined modification can integrate the advantages of different technologies: while significantly enhancing the functional properties of IDF (e.g., hydration properties, adsorption capacity, and prebiotic activity), it effectively addresses issues potentially associated with single modification methods, such as insufficient efficiency, limited specificity, or chemical contamination. Notably, it exhibits broad prospects, especially in fields like intestinal health, metabolic regulation, and food processing.

#### 3.4.1. Physical Methods Combined with Physical Methods

The combined application of two or more physical methods can fully degrade macromolecules in raw materials into small molecules, thereby increasing the content of soluble components. For example, Ullah et al. [44] investigated the effect of heat treatment during wet grinding on the physicochemical properties of black soybean okara insoluble dietary fiber (IDF). The results showed that heat treatment effectively improved the grinding efficiency of black soybean okara IDF in wet grinding; at 120 °C, the modified IDF exhibited the highest grinding efficiency and smallest particle size, with simultaneous improvements in properties such as color, morphology, hydration capacity, stability, and viscosity. In addition to thermo-mechanical combinations, energy-based combined technologies such as ultrasonic-microwave have also shown potential. The cavitation effect of ultrasound can instantly disrupt fiber walls, while the rapid bulk heating of microwaves promotes the vaporization and explosion of internal moisture. Their synergistic effect leads to the drastic loosening of the fiber structure.

#### 3.4.2. Combining Physical Methods with Chemical Methods

By altering the physical structure of dietary fiber via physical pretreatment (e.g., increasing porosity, reducing crystallinity) and subsequently combining with chemical modification, the modification effect can be significantly enhanced. For example, Qi et al. [45] prepared rice bran insoluble dietary fiber (IDF) by combining homogenization with sulfuric acid (H_2_SO_4_) and potassium hydroxide (KOH): First, defatted rice bran was homogenized in boiling sulfuric acid solution, then further treated in boiling potassium hydroxide solution, and subsequently washed until the pH reached neutrality (7.0). The modified rice bran IDF exhibited higher porosity, glucose adsorption capacity (GAC), specific surface area, and α-amylase inhibitory activity.

In addition, ultrasound-assisted chemical modification is an extremely effective combination. The microjets generated by ultrasonic cavitation can greatly enhance the mass transfer efficiency of chemical reagents (e.g., acids, alkalis) into the fiber interior and expose more reaction sites.

#### 3.4.3. Combination of Physical Methods and Biological Methods

The combination of physical and biological methods typically refers to the integration of physical treatment with enzymatic modification. This combination usually involves using physical treatment to create a more favorable environment for bioenzymatic hydrolysis, serving as a model for achieving green and efficient modification. Its synergistic mechanism lies in the fact that physical force fields (e.g., extrusion, ultrasound, and high hydrostatic pressure (HHP)) can disrupt the crystalline regions of insoluble dietary fiber (IDF), increase the specific surface area, and loosen the cell walls, thereby significantly improving the accessibility of enzymes to substrates.

For example, Song et al. [46] investigated the effects of extrusion puffing combined with cellulase on the physicochemical, functional, and structural properties of bamboo shoot dietary fiber (DF). The results showed that the combined extrusion puffing-cellulase treatment altered the structure of bamboo shoot DF without damaging its main components. Compared with single extrusion puffing treatment, the modified bamboo shoot DF exhibited significant improvements in water-holding capacity (WHC), oil-holding capacity (OHC), swelling capacity (SC), glucose adsorption capacity (GAC), cholesterol adsorption capacity (CAC), and nitrite adsorption capacity (NIAC). Similarly, high hydrostatic pressure (HHP) pretreatment can disrupt hydrogen bonds and hydrophobic interactions through hydrostatic pressure, loosening the compact cellulose microfibril structure. This allows enzyme molecules to more easily access and catalyze hydrolysis, making HHP often used to improve the yield of dietary fiber.

#### 3.4.4. Bio-Biological Combined Modification

The combined application of two or more enzyme preparations with different substrate specificities enables the systematic and precise degradation of the complexly intertwined polysaccharide network in plant cell walls, which is difficult to achieve by single enzymatic methods. The combined use of cellulase and xylanase is a common approach in bio-biological combined modification. He et al. [47] pointed out that compared with ultrasound-assisted enzymatic treatment, the combination of cellulase and xylanase exhibited better performance in improving the yield of soluble dietary fiber (SDF) and glucose adsorption capacity (GAC) of rose pomace insoluble dietary fiber (IDF). The underlying mechanism is as follows: First, xylanase degrades the hemicellulose wrapping the outer layer of cellulose microfibrils, eliminating its “shielding effect” on cellulose; then, cellulase can more effectively attack the exposed cellulose backbone, thereby achieving a more thorough degradation effect. In addition, the combination of pectinase and cellulase is also extremely common. Pectinase first disintegrates the pectin matrix in intercellular layers and primary cell walls, creating a breakthrough for subsequent enzymes acting on cellulose and hemicellulose.

### 3.5. Comparative Analysis of Multi-Scale Structural Remodeling of Dietary Fiber Modification

In the field of food science and nutritional health, the structural modification of dietary fiber is a key technical path to enhance its functional characteristics and expand its application scenarios. Techniques such as physical modification, chemical modification, enzymatic hydrolysis, and composite modification enable multi-scale regulation of the dietary fiber’s carbohydrate backbone—from molecular chains and crystal conformations to porous networks and the distribution of surface functional groups. These modifications not only involve molecular structure remodeling, such as glycosidic bond breaking/cross-linking and functional group introduction, but also are accompanied by changes in crystallinity, regulation of porosity and specific surface area, conversion of surface hydrophilicity and hydrophobicity, and re-arrangement of intermolecular forces. Based on the review of relevant literature on the influence of modification methods on the properties of dietary fiber in recent years and the summary, the specific action rules of different modification methods at the levels of molecular structure, crystal structure, pore structure, surface properties and intermolecular interactions can be summarized in Table 2.

## 4. Applied Prospects of Modified Insoluble Dietary Fiber

**Figure 4:**
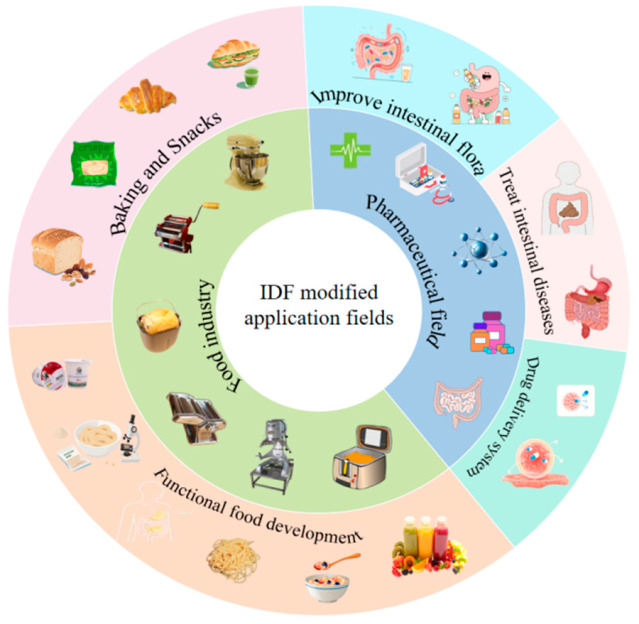
Application fields of modified insoluble dietary fiber.

With the rising consumer demand for low-glycemic and low-fat foods and the urgency for gut health intervention in the aging society, the value of modified IDFs in the food and pharmaceutical industries is becoming more and more prominent. From low GI staple food for regulating blood glucose and probiotic carriers for improving intestinal function, to slow-release drug delivery systems and metabolic disease interventions based on intestinal flora regulation in the pharmaceutical field, modified IDFs have shown a broad prospect for interdisciplinary applications. Based on the research intensity of the core application scenarios, the distribution characteristics of each field can be systematically presented by the following circle diagram (Figure 4).

### 4.1. Food Industry

#### 4.1.1. Application of Modified Dietary Fiber in Functional Foods

Modified dietary fiber (MDF) holds broad application prospects in functional foods. With the growing emphasis on healthy diets among the public, functional foods have gradually become an important product category in the market. Not only do these foods provide basic nutrients, but they also offer additional functions such as disease prevention and health promotion [54]. As a core functional ingredient, modified insoluble dietary fiber (IDF) has been widely incorporated into various functional foods, demonstrating significant effects in enhancing nutritional value, maintaining intestinal health, and exerting hypoglycemic and hypocholesterolemic activities.

In terms of blood glucose regulation, modified insoluble dietary fiber (IDF) effectively stabilizes postprandial blood glucose fluctuations through multiple mechanisms, such as delaying glucose diffusion, adsorbing glucose, and inhibiting starch digestive enzyme activity. This is particularly important for the dietary management of diabetic patients. Studies have shown that modification can significantly enhance the glucose adsorption capacity (GAC) of dietary fiber, with an increase ranging from 28% to 50% [55]. For example, although no direct chemical modification was performed in the study by Wang et al. [56], insoluble and soluble dietary fiber from kiwifruit were applied to a high-fat diet(HFD) combined with streptozotocin (STZ)-induced type 2 diabetes mellitus (T2DM) rat model via dietary supplementation. The results showed that supplementation with both types of dietary fiber significantly improved glycolipid metabolic disorders in rats by regulating gut microbiota (e.g., increasing the abundance of Lactobacillus and Bifidobacterium), reducing fasting blood glucose (FBG), improving insulin resistance (IR), and decreasing body weight. This demonstrates great potential in the development of functional foods for adjuvant treatment of diabetes.

Xie et al. [57] investigated the effects of cellulase hydrolysis of okara combined with extrusion modification on noodle quality, with a focus on optimizing the ratio of soluble dietary fiber (SDF) to insoluble dietary fiber (IDF). After modification, the water-holding capacity (WHC) of IDF increased by 30%, the hydrogen bond interactions of gliadin were enhanced, the hardness of noodles decreased by 25%, and the in vitro starch digestibility decreased by 18%. This dietary fiber modification technology is suitable for the development of low glycemic index (GI) staple foods.

Furthermore, the ability of dietary fiber treated by physical modification technologies such as dynamic high-pressure microfluidization (DHPM) to inhibit glucose diffusion can be increased by more than 62%. In terms of digestive enzyme inhibition, specific modification methods can enhance the inhibition rate of dietary fiber against α-amylase from 44% to 90%. Clinical studies have also provided evidence for this: daily intake of 25 g pea fiber for four consecutive weeks significantly reduced the area under the blood glucose curve (AUC) in overweight or obese individuals. These quantitative data collectively indicate that modified dietary fiber has clear application potential in formulating low glycemic index (GI) foods, such as meal replacement powders and low-GI staple foods [58].

In addition to blood glucose control, modified dietary fiber also plays an important role in promoting intestinal health. Its water-holding capacity (WHC) and swelling capacity can generally be increased by 30% to 50% after modification, which helps enhance satiety and promote intestinal peristalsis. Certain modification methods, especially biological fermentation and chemical modification, can significantly increase the content of soluble dietary fiber (SDF) in dietary fiber, with some reports indicating that the SDF yield can even exceed 60%. This SDF serves as an important nutrient source for beneficial gut microbiota, capable of promoting the production of short-chain fatty acids (SCFAs) (e.g., butyric acid) and the proliferation of beneficial bacteria (e.g., Akkermansia), thereby improving intestinal microecological balance. Furthermore, the cholesterol adsorption capacity of modified dietary fiber is also significantly enhanced, with reported increases ranging from 32% to 90%, providing a basis for its application in lipid-lowering functional foods [59].

In summary, through targeted modification technologies, multiple health-promoting functions of dietary fiber can be significantly enhanced. This enables modified dietary fiber to serve as a key ingredient, widely used in the development of various functional foods such as those promoting intestinal health, regulating blood glucose and lipids, to meet the health needs of specific populations.

#### 4.1.2. Applications in Baking and Snacking

The application of modified dietary fiber (MDF) in baked and snack foods is gradually becoming an important development trend in the food industry, especially against the backdrop of the growing demand for healthy diets and low-calorie products. Modification not only improves the application properties of dietary fiber in baked and snack foods but also endows these products with superior nutritional value and health benefits [60].

In baked foods, modified dietary fiber is mainly added as an ingredient to products such as bread, biscuits, and cakes. It can effectively enhance the nutritional density and dietary fiber content of products without significantly altering their flavor and texture. For example, Prasadi et al. [61] investigated the effects of soluble dietary fiber (SDF) extracted from barley on the rheological properties of wheat dough and baking quality. The results showed that the addition of 2% barley SDF extended the dough stability time from 4.5 min to 7.8 min, significantly reduced the hardness of bread by approximately 25%, and simultaneously improved the specific volume and internal structure of bread. The application of this modified dietary fiber effectively increases the nutritional density of bread, making it more in line with the demand for healthy diets.

However, insoluble dietary fiber (IDF) may cause problems such as increased dough hardness and decreased extensibility during baking. It is necessary to modify IDF through technologies such as physical modification, chemical modification, or enzymatic treatment to improve its hydration capacity, solubility, and viscosity [62].

The effect of adding modified dietary fiber (MDF) to baked foods is relatively complex. Appropriate addition of specific modified dietary fiber helps improve the water-holding capacity (WHC) and stability of dough. For example, chemical modification (e.g., carboxymethylation) can increase the content and water solubility of soluble dietary fiber (SDF), thereby improving the volume and softness of cakes. Some studies have also found that modified dietary fiber can inhibit starch retrogradation and enhance the thermal stability of the system. However, excessive addition or inappropriate modification—especially for insoluble dietary fiber (IDF)—can significantly interfere with the formation of the gluten protein network. Specific manifestations include shortened dough stability time, increased degree of softening, leading to reduced volume, hardened texture, uneven internal air cell structure, and even collapse of products such as bread and steamed buns. A 2025 study by the Chinese Academy of Sciences (CAS) revealed the mechanism underlying the distinctly different effects of highland barley β-glucan (a type of soluble fiber) in bread and steamed buns: it not only inhibits gluten network formation but also the steaming process (high temperature and high humidity) exacerbates this disruption more significantly than baking, resulting in severe collapse of steamed bun structure. This indicates that the impact on product quality is not only related to the fiber addition level but also closely associated with fiber type, processing method, and interactions with other components.

In the field of snack foods, the application of modified dietary fiber aims to enhance health value while maintaining a pleasant sensory experience. Modified dietary fiber can be used in products such as biscuits, potato chips, and nut snacks. Its functions include increasing dietary fiber content, improving crispness, and serving as a fat replacer. Due to its water-holding capacity, gelling properties, and adjustable rheological characteristics, dietary fiber can act as a fat replacer. These fiber-based fat mimics can be categorized into two main types: hydrogels and particle systems, for instance, which simulate the lubricating and rich texture of fat by binding water. They include fiber-stabilized emulsions, emulsion gels, and oleogels. For example, a study constructed oleogels based on natural insoluble soybean fiber, utilizing capillary forces. These systems can reduce total fat and saturated fat content while partially retaining the sensory and functional properties of oils. Achieving these benefits requires precise regulation to avoid adverse effects on product flavor, color, and texture.

The application of modified dietary fiber (MDF) is also extensive in the field of snack foods, especially suitable for processed snack products such as biscuits, potato chips, and nut snacks [63]. Mendes et al. [64] applied green banana flour treated by hydrothermal treatment to the production of gluten-free cupcakes. The results showed that the modified green banana flour not only increased the dietary fiber content of the product but also improved the textural properties of the cupcakes, making them more in line with the development trend of low-calorie and high-fiber healthy snacks. As another important approach, chemical modification mainly includes esterification (e.g., acetylation), etherification (e.g., carboxymethylation), and cross-linking modification. These methods optimize the functional properties of fibers by altering their molecular structures. With the popularization of healthy eating concepts, traditional high-sugar and high-fat snacks are gradually being replaced by healthy snacks characterized by low fat, low calorie, and high dietary fiber.

In summary, the application of modified dietary fiber (MDF) in baked and snack foods is a balancing act. Its successful application relies on the precise control of the addition level and the modification method: identifying the optimal addition level for specific products is crucial. It also requires an in-depth understanding of the interactions between fiber properties and food matrices, including considerations of fiber type (soluble vs. insoluble), modification method, and their complex interactions with components such as gluten and starch. Different processing conditions (e.g., baking vs. steaming) significantly affect the final performance of MDF. Future research will focus more on developing sustainable modification technologies (e.g., enzymatic and physical treatments), exploring the synergistic effects of multi-component composite systems (e.g., protein-fiber complexes), and formulating precision nutrition strategies based on nutrigenomics and microbiome science. Through interdisciplinary collaboration, modified dietary fiber is expected to achieve a better balance between health and palatability, driving the innovation of next-generation healthy foods.

### 4.2. Medical Field

#### 4.2.1. Application of Insoluble Dietary Fiber in Drug Delivery Systems

Dietary fiber, as a drug carrier, exhibits excellent biocompatibility and biodegradability, making it a safe and environmentally friendly drug delivery material. After modification, insoluble dietary fiber (IDF) can regulate its physical properties, such as swelling capacity, water absorption, and adhesiveness, thereby achieving a sustained drug release effect.

For example, Yang et al. [65] used chemical modification technology to carboxymethylate dietary fiber extracted from wheat straw, preparing a polyelectrolyte composite hydrogel that was applied in long-acting gastric retention and drug delivery systems. Studies have shown that this hydrogel, based on modified dietary fiber derivatives, possesses outstanding swelling performance and mechanical stability. It can effectively control the release rate of model drugs (e.g., 5-fluorouracil (5-FU)) in simulated gastric fluid (SGF), achieving sustained release for up to 24 h. Chemical modification technologies can endow insoluble dietary fiber (IDF) with a more stable and dense structure, enabling it to effectively retard the in vivo drug release rate. In terms of targeted drug delivery, modified IDF can achieve drug release at specific targets through conjugation with other targeted drug molecules or antibodies. Through enzymatic or microbial fermentation modification, the biodegradability of dietary fiber can be further enhanced, allowing for targeted drug release in specific biological microenvironments [65].

Enzymatically modified insoluble dietary fiber (IDF) exhibits higher biodegradability and can control the release of anticancer drugs in specific environments. For example, Bakr et al. [66] systematically discussed the mechanism of action of soluble dietary fiber (SDF) as an antihyperlipidemic agent in their review, emphasizing that physical, chemical, or enzymatic modification (e.g., enzymolysis) can enhance its ability to bind bile acids and cholesterol, thereby exerting a targeted lipid-lowering effect in the intestines. Su et al. [67] reviewed the application of nanotechnologies such as nanoemulsions and nanocapsules in IDF modification: particle size regulation improves the dispersibility and bioavailability of IDF. In addition, nanostructured IDF can load probiotics or antioxidants for the development of pH-responsive beverages, achieving intestinal targeted release. Furthermore, the integrated application of nanotechnology expands its functional boundaries: by constructing nanoemulsions or nanocapsules, the dispersibility and bioavailability of IDF can be improved. Nanostructured IDFs can not only load small-molecule drugs but can also encapsulate probiotics or antioxidants, which are used to develop pH-responsive smart beverages enabling intestinal targeted delivery [67].

However, modified insoluble dietary fiber (IDF) still faces several key challenges and limitations in practical applications. First and foremost, there is high variability in its biodegradability: the degradation rate is influenced by multiple factors, such as fiber source, modification method, and interindividual differences in gut microbiota, which pose challenges to achieving consistent and predictable drug release kinetics. Second, there is a complex interaction between drug release and human gastrointestinal transit time; due to the limited residence time of drugs in the gastrointestinal tract and significant individual variability, if the fiber carrier degrades too slowly, it may result in incomplete drug release before excretion, thereby reducing its bioavailability. Furthermore, as a macromolecular material, modified IDF’s drug-loading efficiency, compatibility with drugs of different chemical properties, and process reproducibility in large-scale production are also technical bottlenecks that need to be addressed in practical applications.

#### 4.2.2. Potential of Modified Dietary Fiber in the Treatment of Intestinal Diseases Such as Constipation

After appropriate modification, modified dietary fiber (MDF) can not only improve its own solubility, water absorption capacity, and swelling capacity but also enhance its functional effects in the intestines, thereby effectively alleviating constipation, regulating intestinal microecological balance, and promoting intestinal health. In terms of constipation alleviation, its mechanism not only relies on the traditional “water absorption and swelling” theory but is also closely related to the fiber’s water-holding capacity (WHC), physical stimulation of intestinal motility, and microbial fermentation products.

For example, Zhang et al. [68] conducted a comparative study on the alleviating effects of five soluble dietary fibers (SDFs) with different sources and structural characteristics on loperamide-induced constipation in mice. The results found that the physicochemical structure of dietary fiber (e.g., molecular weight, monosaccharide composition, and WHC) is the key determinant of its efficacy. Studies have shown that dietary fibers with high WHC and viscosity (e.g., glucomannan derived from konjac) can more effectively absorb water and swell, increase fecal volume and water content, and significantly shorten intestinal transit time, thereby achieving better improvement in constipation symptoms.

Furthermore, the intestinal microecological regulatory function of modified dietary fiber has also been verified by clinical studies. Specific modification processes (e.g., enzymatic treatment) can partially convert insoluble dietary fiber (IDF) into substrates preferentially utilized by gut microbiota. For example, Zhu et al. [69] used the complex enzymatic method (cellulase and papain) to extract soluble dietary fiber (SDF) from sunflower heads, and evaluated its alleviating effect on constipation and regulatory effect on gut microbiota in mice. The results showed that this SDF could effectively shorten the first black stool time of mice, increase fecal water content, and significantly enhance intestinal peristalsis capacity. In terms of gut microbiota regulation, SDF intervention significantly increased the relative abundance of beneficial bacteria (e.g., Lactobacillus and Bifidobacterium), while reducing the Firmicutes/Bacteroidetes (F/B) ratio. This effectively improves intestinal dysbiosis caused by constipation and restores intestinal microecological balance.

Notably, dietary fibers obtained via different modification methods exhibit variations in their physiological effects. Physical modification (e.g., ultrafine grinding) primarily enhances water-holding capacity by increasing the fiber’s specific surface area, thereby accelerating intestinal transit. In contrast, enzymatic modification alters the molecular structure of fibers to improve their fermentability, enabling more precise regulation of gut microbiota composition. Therefore, when developing functional foods targeting intestinal health, it is necessary to select fiber raw materials with corresponding modification processes based on specific health objectives (e.g., constipation alleviation or microbiota regulation).

In summary, the application value of modified dietary fiber (MDF) in relieving constipation and regulating gut microbiota has been supported by clinical human trials. Future research should continue to focus on the long-term intervention effects of different modified fibers in specific populations (e.g., different age groups or disease states), so as to promote their precision application in personalized nutrition and clinical dietary support.

#### 4.2.3. Research on Modified Dietary Fiber in Improving Gut Microbiota

In recent years, through structural optimization and functional enhancement, modified dietary fiber (MDF) has demonstrated significant potential in regulating gut microbiota and improving host metabolic health. Studies have shown that corn bract fiber modified by dynamic high-pressure microfluidization (DHPM) can effectively reduce weight gain and insulin resistance (IR) in obese mice. Its mechanism of action is closely related to promoting the proliferation of Bifidobacterium, inhibiting the excessive proliferation of Firmicutes, and significantly increasing the production of short-chain fatty acids (SCFAs)—especially the elevation of butyrate levels, which exerts a protective effect on maintaining intestinal barrier integrity [70]. Similarly, tartary buckwheat bran fiber treated by steam explosion technology exhibited dual regulatory effects in diabetic model mice: it reduced gluconeogenesis by activating the hepatic AMPK pathway, while promoting colonic SCFA production and activating the GPR43/GLP-1 axis, thereby achieving the maintenance of glucose homeostasis [71].

For example, Lamothe et al. [72] found that after microwave treatment of pearl millet insoluble dietary fiber (IDF) at 600 W for 5 min, followed by complex enzymolysis with α-amylase and amyloglucosidase, its fermentability was significantly enhanced. In vitro fermentation experiments showed that the butyrate production of the modified fiber doubled, and acetate production nearly tripled. Additionally, it promoted the proliferation of Firmicutes (e.g., Blautia and Coprococcus) and inhibited the growth of Proteobacteria, indicating its regulatory effect on gut microbiota. Ma et al. [73] systematically discussed the production, structure, biological activities, and applications of tremella polysaccharides in a review. Studies have pointed out that, as a typical soluble dietary fiber (SDF), the biological activities of tremella polysaccharides (e.g., antioxidant, hypoglycemic, and gut microbiota regulatory effects) are closely related to their molecular structure, molecular weight, and monosaccharide composition. Specifically, tremella polysaccharides can be fermented by colonic microbiota, promoting the growth of beneficial bacteria (e.g., Bifidobacterium and Lactobacillus) and stimulating the production of short-chain fatty acids (SCFAs). These changes in biological activities are associated with the effects of dietary fiber modification on human health, providing a biological activity-based basis for evaluating the modification effect of dietary fiber. Dai et al. [74] studied chemically modified okara IDF and found that it could significantly reduce fat accumulation and serum cholesterol levels in high-fat diet (HFD)-fed mice. This modified IDF improved metabolic disorders by regulating gut microbiota and SCFA metabolism, offering a new perspective for the nutritional intervention of intestinal diseases.

Although insoluble dietary fiber (IDF) modification technologies hold broad prospects, their transition from laboratory-scale research to large-scale application still faces a series of core challenges. Most modification technologies (e.g., dynamic high-pressure microfluidization (DHPM), steam explosion) exhibit remarkable effects in laboratory settings; however, scaling them up to pilot or industrial production encounters challenges related to equipment throughput, stability of continuous production, and cost control. For instance, high-pressure homogenization equipment is prone to clogging when processing high-viscosity IDF slurries, making it difficult to maintain long-term continuous operation. There are significant differences in operating costs and energy consumption among various technologies: physical field technologies (e.g., ultrasound, microwave) demand high energy inputs, while enzymatic treatment is constrained by the cost of high-purity enzyme preparations. Although composite modification technologies can enhance functional effects, multi-step processing also increases the overall cost and energy consumption. Evaluating the sustainability of these technologies requires a comprehensive consideration of factors such as energy consumption, waste generation, and solvent usage.

Regulatory and Safety Barriers: As a food raw material or ingredient, modified insoluble dietary fiber (IDF) must comply with national regulations. Key considerations include residue limits for food-grade chemical modifiers (e.g., esterification and etherification reagents), lists of permitted enzyme preparations, and the compliance of novel physical processing technologies (e.g., high-pressure homogenization, pulsed electric field (PEF)). The overall environmental footprint of the modification process has attracted increasing attention. An ideal modification technology should feature low waste generation, solvent recycling, and by-product valorization. For example, supercritical CO_2_ fluid modification technology is regarded as a green alternative.

Currently, modified IDF research faces the challenge of inconsistent characterization methods. There is a lack of unified, standardized protocols for evaluating the structure (e.g., crystallinity, porosity), functional properties (e.g., water-holding/oil-holding capacity, swelling capacity), and final physiological effects of modified IDF, resulting in poor comparability of data among different studies. Establishing a consistent characterization and in vitro evaluation system is crucial for advancing scientific research and industrial applications in the field of IDF modification.

Modified IDF holds enormous potential for improving host health by regulating gut microbiota. Future research should focus on: developing low-cost, high-efficiency, and scalable modification technologies; strengthening the safety and compliance assessment of modified IDF; establishing a unified structure–function–physiological effect characterization system; and conducting in-depth clinical studies on precise and personalized modified IDF products targeting specific health issues.

## 5. Challenges and Prospects of Insoluble Dietary Fiber Modification Technology

### 5.1. Technical Bottlenecks and Optimization of Modification Effects

Although the modification technology of insoluble dietary fibers shows great potential in the fields of food and medicine, they still encounter several technical bottlenecks in practical applications. Firstly, the efficacy of modified dietary fibers is often limited by the selection of modification methods and operating conditions. For example, while heat treatment can improve the swelling capacity of dietary fibers, excessively high temperatures or prolonged durations may cause nutrient degradation or structural damage. Similarly, chemical modification methods such as esterification and oxidation treatments, while significantly altering the solubility and functionality of dietary fibers, may introduce harmful chemical residues that affect the safety and health of the final product.

Secondly, dietary fibers derived from diverse sources exhibit varying responses to modification, complicating the standardization and optimization of the modification processes. The molecular structure, sources and physicochemical properties of dietary fiber vary greatly, resulting in inconsistent modification effects among different types of dietary fiber. Therefore, devising tailored modification strategies and precisely regulating them based on the specific characteristics of various dietary fibers has become a crucial focus for enhancing modification efficacy.

To overcome these challenges, future research should focus on the optimization and intelligent control of the modification process. For instance, by optimizing modification parameters, developing eco-friendly and safe modification techniques (such as biological methods), and deepening research into the diversity of dietary fiber sources, the effectiveness and application range of dietary fiber modifications can be significantly improved.

### 5.2. Market Prospects and Development Trends of Modified Dietary Fiber

With growing public awareness of healthy diets and disease prevention, the market prospects for modified dietary fiber are expanding rapidly. Dietary fiber, especially modified dietary fiber, is gradually becoming an important ingredient in functional foods, healthcare products and drug delivery systems due to its potential in improving intestinal health, controlling blood glucose and lowering cholesterol. As consumers’ requirements for nutritional value and functionality of food increase, the market demand for modified dietary fiber continues to grow.

In the food industry, modified dietary fiber can not only improve the taste and solubility of traditional dietary fiber, but also enhance its functionality, and is widely used in high-fiber foods, weight loss foods, functional beverages and other fields. With the changes in people’s dietary structure, especially the prevalence of high-sugar and high-fat diets, the incidence of chronic diseases such as constipation, obesity and diabetes is constantly rising. Modified dietary fiber, as a natural and safe dietary supplement, is playing an increasingly important role in the prevention and treatment of these diseases. Consequently, the modified dietary fiber market is expected to maintain a steady growth in the coming years.

In the field of medicine, the application prospects of modified dietary fiber in drug delivery systems are also very broad. Owing to its excellent biocompatibility and controllable release characteristics, it has been utilized in sustained-release formulations, enteric-coated preparations, and other drug delivery vehicles, significantly enhancing drug efficacy and patient compliance. In addition, with the increasing concern over intestinal health issues, the potential of modified dietary fiber in treating constipation, intestinal inflammation and other diseases has been continuously verified, and it is expected to demonstrate greater market value in the field of intestinal disease treatment.

With ongoing advancements and innovations in modification technology, modified dietary fiber is expected to unlock broader application opportunities in the future. Scientific and technological progress will promote the transition of modified dietary fiber from traditional physical and chemical modification to more environmentally friendly and green biomodification technology. In addition, intelligent and personalized dietary fiber products will also become a development trend. By precisely regulating the functional properties of dietary fiber, the development of products more in line with consumer demand will further promote its market expansion.

## 6. Economic Feasibility Analysis of Dietary Fiber Modification: Market Expansion and Cost Optimization Driven by Technological Breakthroughs

Modified dietary fiber (MDF) technologies have demonstrated significant economic feasibility by enhancing product added value, expanding application scenarios, and reducing production costs. The core logic lies in the two-way drive between technological breakthroughs and market demand. Below is an analysis from three dimensions—cost-effectiveness, market premium, and industrial chain synergy—supported by specific data and cases.

### 6.1. Modified Technologies Reduce Production Costs and Improve Raw Material Utilization Rate

Traditional dietary fiber extraction processes suffer from low yield and insufficient purity. In contrast, modified technologies significantly improve raw material utilization rate and reduce unit costs by optimizing extraction and purification processes. For example, enzymolysis technology: In the extraction of dietary fiber from potato pomace, the adoption of a combined enzymolysis process with α-amylase and cellulase increased the dietary fiber yield from 55% to 83.65%. Meanwhile, it reduced the usage of acid–base reagents and lowered wastewater treatment costs. According to research, the enzymolysis process decreased the production cost of resistant dextrin from 32,000 yuan/ton in 2019 to 18,000 yuan/ton in 2024, promoting the popularization of downstream applications. Physical modification: Ultrafine grinding technology improves dietary fiber solubility and reduces processing loss by decreasing particle size (e.g., from 100 μm to 10 μm). Taking baked foods as an example, the addition level of modified dietary fiber can be increased from 5% to 10%, while the dough rheological properties are improved, and the product qualification rate is raised by 15%, indirectly reducing production costs.

### 6.2. Modified Products Achieve Market Premium and Expand High-Value-Added Fields

By enhancing functionality and optimizing taste, modified dietary fiber (MDF) meets consumers’ dual demands for health and quality, thereby gaining significant market premium space. Functional foods: The application of modified dietary fiber in dairy products and beverages has significantly increased product value. For example, high-fiber yogurt fortified with micronized dietary fiber (with solubility increased by 30%) has a unit price 40% higher than that of ordinary yogurt, and its repurchase rate has risen by 25%. In 2023, the market size of functional dairy products in China reached 12 billion yuan, of which dietary fiber-based products accounted for over 30%.

Medical foods: The addition level of modified dietary fiber in enteral nutrition preparations has increased from 5% to 12%, meeting the nutritional needs of post-surgical patients. In 2023, the market size of the medical food segment reached 580 million yuan, with an annual growth rate exceeding 20%, where modification technologies serve as the core driving force.

Through cost optimization, functional enhancement, and market expansion, dietary fiber modification has formed a clear profit path. Technological breakthroughs (e.g., enzymolysis, micronization, fermentation) and policy support (e.g., the “Healthy China 2030” Initiative) have jointly promoted the industry’s transformation from “concept popularization” to “scientific application.” In the future, as modification technologies evolve towards nanosization and intelligence, dietary fiber will unlock greater value in emerging fields such as precision nutrition and 3D-printed foods, with sustained improvement in economic feasibility. Enterprises need to focus on technological iteration and scenario deepening, seizing market opportunities with differentiated products to achieve sustainable development.

## 7. Conclusions and Future **Perspectives**

Research on insoluble dietary fiber (IDF) modification has evolved from simple functional enhancement to a systematic science integrating multi-scale structural regulation and precision functional design. This review reveals that physical, chemical, and biological modification technologies can directionally enhance the hydration properties, adsorption capacity, and fermentability of IDF by synergistically disrupting the fiber’s crystalline regions, increasing its specific surface area, and introducing active functional groups. The common core mechanism underlying these effects lies in the precise regulation of the fiber’s “structure–function–physiological effect” relationship.

However, modified dietary fiber (MDF) still faces several challenges in technical application, such as the instability of modification effects and the loss of nutrients during the modification process. Future research needs to optimize modification processes, improve modification efficiency, and ensure product safety, while focusing on the standardization and operability of technologies to promote the industrial application of MDF.

Key challenges that urgently need to be addressed in future research include: promoting the transformation of modification strategies from generalization to precision and personalization, and establishing customized schemes based on raw material characteristics and target functions; developing green and low-energy-consuming composite modification technologies, and systematically evaluating their scalability, cost-effectiveness, and environmental impact; establishing international standards for the characterization of IDF structure and function to enhance the comparability and translational value of research data; deepening human studies and integrating microbiomics and metabolomics to reveal the mechanism of action of IDF in real physiological environments; exploring innovative applications of IDF in precision nutrition and targeted delivery, and expanding its boundaries as an intelligent food ingredient and drug carrier. Through interdisciplinary collaboration and methodological innovation, IDF modification technology is expected to achieve greater breakthroughs in the fields of healthy foods and biomedicine.

## Figures and Tables

**Table 2:** Summary of the effects of different modification methods on the properties of dietary fiber.

Modification Method	Molecular Structure	Crystal Structure	Pore Structure	Surface Properties	Intermolecular Interaction	Reference
Heat treatment	Molecular chain breaks, glycosidic bond breakdown, molecular weight ↓	Crystallinity ↓, amorphous zone ↑	Porosity ↑, Specific surface area ↑	Roughness ↑, hydrophilicity ↑	Hydrogen bonds break, van der Waals force ↓	[48]
Alkaline treatment + enzymatic modification	hemicellulose is removed and cellulose is exposed; enzymatic digestion produces short chains	Crystallinity ↓, X-ray diffraction peaks weakened	Microporous/mesoporous ↑, porous network formation	Polar groups are exposed and zeta potentials are more negative	Hydrogen bonding reduced, hydrophobic interactions ↑	[49]
Extrusion/Chemical Modification	Rearrangement of molecular chains, introduction of new bonds by cross-linking agents	Crystallinity ↓, crystal size decrease	Porosity ↑, pore size distribution shifted to large pores	Deepening of surface grooves, hydrophilicity ↑	Alternating hydrogen bonding/hydrophobic interactions	[46]
Chemical modification	Introduction of functional groups such as ester group/-COOH	Crystallization zone disrupted, amorphous structure ↑	Cross-linking leads to ↓ porosity and structural densification	Polarity ↑ or hydrophobicity ↑	New chemical bond (ester bond) formed, hydrogen bond ↓	[50]
γ-irradiation + enzymatic modification	Free radical-induced glycosidic bond breaking, molecular weight ↓	Crystallinity ↓, lattice distortion ↑	Microporosity ↑, specific surface area ↑ (enzymatic digestion after irradiation)	Surface Polar Groups ↑	Hydrogen bonds broken, hydrophobic interactions ↑	[51]
Probiotic fermentation modification	Fiber decomposition to oligosaccharides, molecular weight ↓	Crystallinity ↓, fermentation products cover surface	Porosity ↑, pore size distribution homogenization	Hydrophilicity ↑, negative surface charge ↑	Hydrogen bonding is reduced and microbial metabolites are cross-linked	[40]
Ultrafine grinding + enzymatic modification	Shortening of molecular chains and exposure of cellulose microfilaments	Crystallinity ↓, crystal arrangement disordered	Nanoscale pore formation, specific surface area ↑	Roughness ↑, Hydroxyl Exposure ↑	Hydrogen bonding is reduced and mechanical stresses lead to structural relaxation	[52]
Non-thermal modification (ultrasonic/high-pressure treatment)	Molecular chain breaks, glycosidic bond rearrangements	Crystallinity ↓, amorphous structure ↑	Porosity ↑, pore size distribution shifted towards mesopores	Hydrophilicity ↑, negative surface charge ↑	Hydrogen bonds break and van der Waals forces rearrange themselves	[23]
chemical treatment	hemicellulose removal, cellulose purity ↑	Crystallinity ↓, cellulose I → cellulose II transition	Porosity ↑, increased pore connectivity	Polar groups ↑, surface charge density ↑	Hydrogen bonding reduced, hydrophobic interactions ↑	[39]
Enzymatic + Extrusion Cooking	Molecular chain breakage, starch pasting	Crystallinity ↓, amorphous starch-coated fibers	Porosity ↑, porous sponge-like structure	Hydrophilicity ↑, starch coating increases adhesion	Hydrogen bonding reduced, starch-fiber interactions ↑	[53]

An upward arrow indicates an increase, and a downward arrow indicates a decrease.

## Data Availability

No new data were created or analyzed in this study. Data sharing is not applicable to this article.

## Abbreviations

DF	Dietary Fiber
IDF	Insoluble Dietary Fiber
SDF	Soluble Dietary Fiber
UD	Uniform design
HPH	High-pressure homogenization
HHP	High hydrostatic pressure
DS	Degree of substitution
AHP	Alkaline hydrogen peroxide
FTIR	Fourier transform infrared spectroscopy
SEM	Scanning electron microscopy
WHC	Water holding capacity
OHC	Oil holding capacity
SC	Swelling capacity
GAC	Glucose adsorption capacity
CAC	Cholesterol adsorption capacity
NIAC	Nitrite ion adsorption capacity
SCFAs	Short-chain fatty acids
